# Determination of the unsaturated disaccharides of hyaluronic acid in equine synovial fluid by high-performance liquid chromatography and fluorescence detection

**DOI:** 10.1186/s13028-015-0098-y

**Published:** 2015-03-04

**Authors:** Kaisa Aaltonen, Tytti Niemelä, Satu Sankari, Riitta-Mari Tulamo

**Affiliations:** Department of Equine and Small Animal Medicine, University of Helsinki, P.O. Box 57, 00014 Helsinki, Finland

**Keywords:** Equine, High-performance liquid chromatography, Hyaluronic acid, Synovial fluid

## Abstract

**Background:**

The purpose of this study was to develop and validate an analytical method to determine the presence of hyaluronic acid derived disaccharides in equine synovial fluid.

**Findings:**

A high-performance liquid chromatography method for the determination of hyaluronic acid derived unsaturated disaccharides in equine synovial fluid was developed and validated. The method is based on the measurement of unsaturated disaccharides released by digestion of linear hyaluronic acid molecules. The method showed linearity (*r*^*2*^ = 0.996) over the full working concentration range 0.89-30 mg/l. Relative standard deviation of intra- and inter-day precision ranged from of 4.3-6.7% and 7.1-7.8% respectively. The detection limit was 0.3 mg/l corresponding to 20 mg/l in synovial fluid. Accuracy of the assay was 97-103%. This method was evaluated by determining the concentration of unsaturated disaccharides from hyaluronic acid in synovial fluid of horses with lameness in the metacarpo-/metatarsophalangeal joint localized with positive response to intra-articular anesthesia.

**Conclusions:**

The described method is valid for determination of hyaluronic acid derived disaccharides in equine synovial fluid. This method was applied to a larger research project dealing with a new form of intra-articular therapy in horses with arthritic diseases.

## Findings

Hyaluronic acid (HA) is a linear polysaccharide formed of disaccharide units which are linked together to form the HA chain. The molecular mass of HA can be as high as 10^7^ Daltons depending on the number of repeating disaccharide units in the chain [1].

The viscoelasticity of synovial fluid (SF) is attributable to its rich HA content and this molecule serves as the principal lubricant of synovial soft tissues [2]. In arthritis, the synthesis of HA is decreased and degradation enhanced. This results in the reduction of HA concentration and molecular mass, and diminished viscoelasticity in SF [3]. The concentration of HA can be used as a diagnostic marker for arthritis where a significantly lower level of HA compared to that of control horses has been reported [4]. The effects of various intra-articular treatments could also be monitored using the concentration of HA in the SF.

Various methods have been described for the estimation of the quantity of HA in SF by using either polymeric or fragmented molecules, such as: size-exclusion chromatography, fluorophore-assisted carbohydrate electrophoresis, enzyme-linked immunosorbent, radiometric binding, and colorimetric assays [5-9]. Analysis of high-molecular mass HA with different degree of polymerization is challenging. The unavailability of size exclusion columns with high exclusion limits causes difficulties in the analysis of very long HA chains. By digestion of HA to unsaturated disaccharides and labelling with a hydrophobic fluorescent agent, the analysis is enabled by reverse-phase high-performance liquid chromatography (HPLC) which is reported to offer good resolution [10,11]. Labeling by a fluorescent molecule also increases detection sensitivity and specificity significantly [12]. Reverse-phase HPLC has been used to analyze HA-disaccharides in biological samples [10], however to our knowledge the method is not validated for equine SF.

The aim of this study was to develop and validate an accurate and specific HPLC method, with low sample volume, for determination of the concentration of unsaturated disaccharides of HA (ΔDi-HA) in equine SF and apply the method as part of a larger study. The indirect method to determine the total amount of HA is based on the analysis of ΔDi-HA produced by digestion. To evaluate the suitability of the method, ΔDi-HA was quantified in SF of the equine metacarpo-/metatarsophalangeal (MCP/MTP) joints.

Twenty eight equine SF samples were analyzed. Samples were obtained by performing routine aseptic arthrocentesis with an 18 gauge/3.8 cm needle which was introduced into the MCP/MTP joint through the lateral sesamoidean ligament. SF sample was withdrawn and 10 ml anesthetic solution, mepivacaine hydrochloride (Scandicain®, Astra Zeneca, Zug, Switzerland) was injected intra-articularly (IA). The horses were free of lameness when evaluated 10 min after IA-anesthesia which verified that lameness was originating from this joint. The joints were medicated and results are reported elsewhere.

SF samples were centrifuged for 10 min at 4000 x *g* at 4°C. Supernatants were removed and frozen at -80°C until analyzed. All samples were analyzed in duplicate.

Before analysis, SF sample was thawed and diluted 1:1 in water. An aliquot (5 μl) was digested with chondroitinase ABC (cABC) from *Proteus vulgaris* (Sigma Aldrich, St Louis, MO, USA) (25 mU) in 50 mM ammonium acetate buffer, pH 6.8 at 37°C for 20 h. After digestion, 900 ng internal standard ΔDi-UA-2S (Sigma Aldrich, St Louis, MO, USA) was added and the mixture was filtrated using a centrifugal filter (Ultrafree-MC centrifugal filter device, Amicon Bioseparations, Millipore, MA, USA) at 12 000 x *g* for 45 min at 4°C. The filtrate was evaporated to dryness in a vacuum evaporator.

Dried disaccharides were derivatized as described previously [13,14]. Briefly, 5 μl of 0.1 M 2-aminoacridone (AMAC) was added in glacial acetic acid / dimethyl sulfoxide (DMSO) (3:17, v/v) and incubated at room temperature for 15 min. Then 5 μl freshly prepared 1 M cyanoborohydride was added and the mixture was incubated at 45°C for 4 h. Finally, 190 μl of 50% (v/v) DMSO was added and 10 μl of clear filtrate was analyzed.

For standards, 1 mg ΔDi-HA (Sigma Aldrich, St Louis, MO, USA) and ΔDi-UA-2S were dissolved in 1 ml 90% methanol separately. Working solutions were made by diluting the stock solutions 1:10 in 90% methanol. Standards were stored at -20°C. ΔDi-HA was taken to yield final concentrations between 0-30 mg/l and ΔDi-UA-2S 4.5 mg/l. The standards were evaporated and derivatized like samples and they were made weekly.

Chromatography was performed on an 1100 series HPLC (Agilent Technologies Co., Waldbronn, Germany) and a fluorescence detector (excitation at 442 nm and emission 520 nm). The column used was a Zorbax Eclipse XDB-C18 (4.6 mm x 250 mm, 5 μm) (Agilent Technologies Co., Little Falls (Wilmington), Delaware, USA) fitted with the guard column of the same type. The flow rate was 1 ml/min. The column was first equilibrated with 98% buffer (100 mM ammonium acetate, pH 5.6) (A) and 2% methanol (B) for 10 min. A linear gradient was run starting with the initial condition for 2 min to 60% of eluent B in 40 min. Finally the column was flushed with 60% eluent B for 9 min. Column temperature was held at 22°C. Total run time was 61 min.

A calibration curve was obtained by plotting peak areas of ten ΔDi-HA standards against their concentrations followed by linear regression analysis calculated using the method of least squares. The peaks were identified by comparing their retention times with those obtained from standards and confirmed by spiking technique. The samples were quantified using the peak area of ΔDi-HA and corrected using the ratio of a peak area of the internal standard in the sample and a peak area of the internal standard in the standard solutions.

Chondroitin sulfate (CS) may be present in small amounts in SF [15]. Chondroitinase ABC degrades HA as well as CS to the unsaturated disaccharides. Nonsulfated CS-disaccharides (ΔDi-0S) may interfere with the analysis of HA-disaccharides by eluting with the same retention time by HPLC whereas sulfated CS-disaccharides separate from the peak of the ΔDi-HA because of decreasing hydrophobicity. The current assay was compared for resolution of CS-disaccharides by digesting chondroitin sulfate sodium salt from shark cartilage (Sigma-Aldrich, St. Louis, MO, USA) by cABC using the same conditions as for SF samples and analyzed with the same method.

The intra-day precision was established by analyzing two equine SF samples five times in one day under the same conditions. The inter-day precision was determined by analyzing these same samples over five different days. The precision was expressed as relative standard deviation (RSD%).

The analytical sensitivity of the method was expressed as the limit of quantification defined as the minimum amount of ΔDi-HA that resulted in a peak height that was ten times that of baseline noise and the limit of detection, which has a peak height three times that of baseline noise.

The accuracy of the method was assessed by spiking two different concentrations of ΔDi-HA in the known equine SF and comparing measured and actual values. The percentage recovery was reported as accuracy.

**Figure 1:**
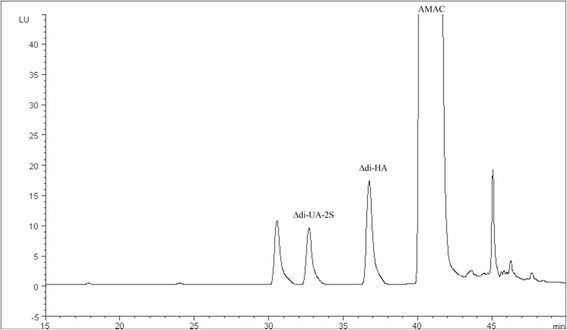
**Chromatogram of AMAC-labelled ΔDi-HA, and internal standard, ΔDi-UA-2S.** Chromatography performed on a reverse-phase column with a linear gradient of 2 - 60% acetonitrile. Disaccharides were detected by fluorescence excitation at 442 nm and emission at 520 nm.

A typical chromatogram is shown in Figure 1. Peaks of ΔDi-UA-2S and ΔDi-HA separated with good resolution. The retention times were 32.1 min and 36.3 min respectively. The CS-disaccharides, fragmented from shark cartilage with cABC, eluted with different retention times. The amount of total CS in equine SF is around 30 mg/l in normal rested and exercised horses and the portion of ΔDi-0S of total CS is shown to be low [15]. In this method, the concentration of ΔDi-0S was not studied because its interference to the concentration of HA-disaccharides is negligible.

The calibration curve for ΔDi-HA standards showed a linear response over the full concentration range (0.89–30 mg/l) with a correlation coefficient *r*^*2*^ = 0.996. The intra-day and inter-day precisions (RSD%) were 4.3–6.7% and 7.1–7.8% respectively. The detection limit was 0.3 mg/l corresponding to 20 mg/l in SF. The limit of quantitation was 0.89 mg/l corresponding to 70 mg/l in SF. The accuracy of the assay was 97-103%.

**Figure 2:**
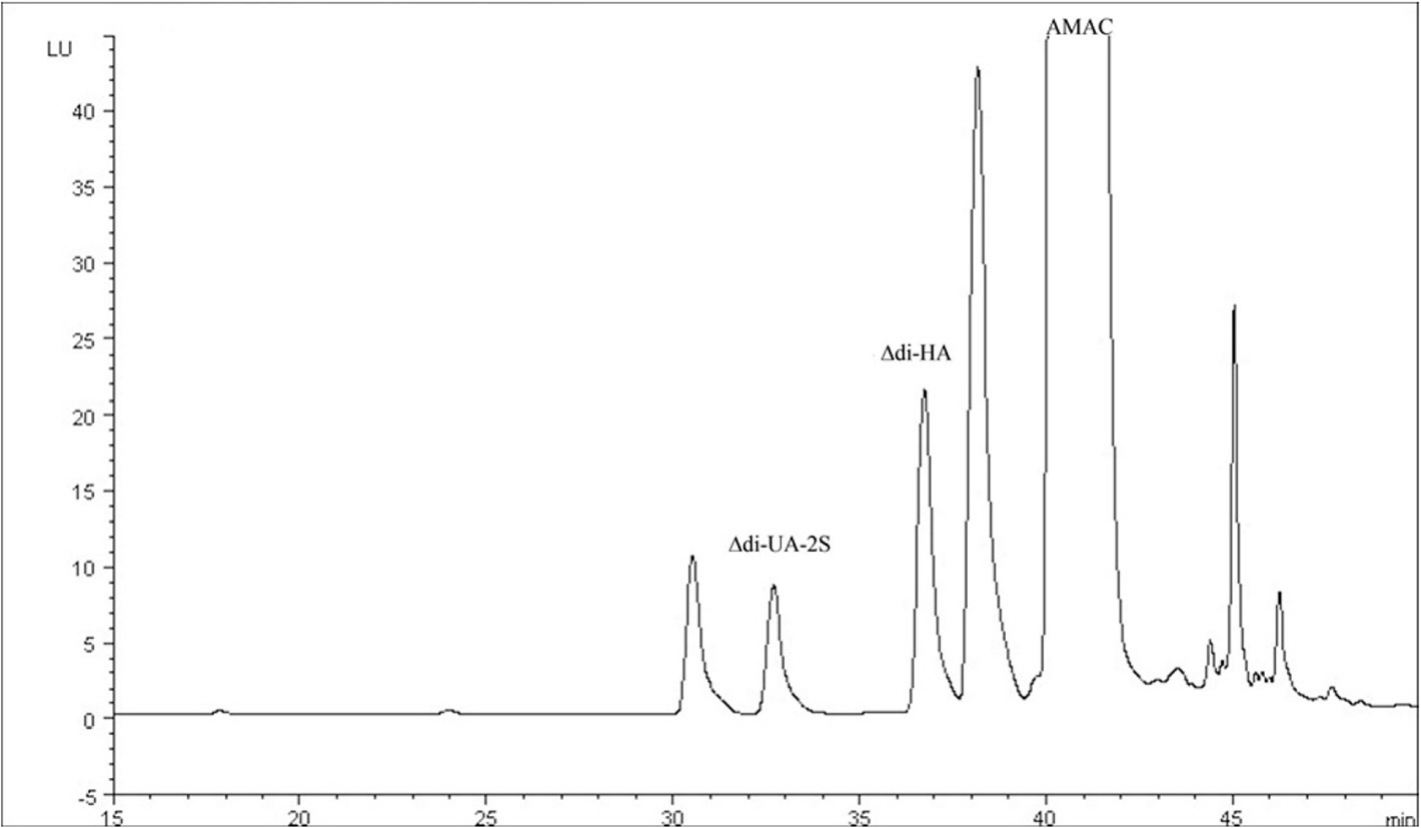
**Typical chromatogram of AMAC-labelled ΔDi-HA fragmented by cABC from hyaluronic acid in SF of the equine metacarpo-/metatarsophalangeal joint and internal standard, ΔDi-UA-2S.**

Figure 2 shows a typical chromatogram of unsaturated disaccharides produced from HA in equine SF. The mean ΔDi-HA concentration (*n* = 28) was 832 mg/l (range 364–1980 mg/l) in SF from the MCP/MTP joint in horses with lameness.

ΔDi-HA has been successfully analyzed by reverse-phase HPLC with pre- [10,11] and postcolumn [16] derivatization. Among others, danzylhydrazine [12], 2-aminopyridine [17], and AMAC [14] have been used as pre-column reagents. The reaction of AMAC with the ΔDi-HA is simple and rapid. Hydrophobic AMAC-labeling permits separation by reverse-phase HPLC eliminating the need for ion-pair reagents [18]. AMAC-derivatives have been shown to be stable for one month at room temperature, for longer periods of time, storage at -80°C is recommended [19].

In summary, the present method for quantifying ΔDi-HA in equine SF was validated for linearity, accuracy, inter-day, and intra-day precision, specificity, detection and quantitation limits. Reverse-phase HPLC used here provides a useful, accurate, and specific method for measurement of the AMAC-labelled unsaturated disaccharides. Because of high content of HA in SF, just a few microliters of SF are required for the present method. Since only a conventional HPLC apparatus with a fluorescence detector is required, this method can be employed in most laboratories. This analytical method was successfully used to monitor disaccharides derived from HA in equine SF.

## Abbreviations

AMAC: 2- aminoacridone
cABC: chondroitinase ABC
CS: chondroitin sulfate
DMSO: dimethyl sulfoxide
HA: hyaluronic acid
HPLC: high-performance liquid chromatography
IA: intra-articularly
MCP/MTP: metacarpo-/metatarsophalangeal joint
RSD: relative standard deviation
SF: synovial fluid
ΔDi-0S: nonsulfated unsaturated disaccharides of chondroitin sulfate, 2-acetamido-2-deoxy-3-*O*-(β-D-gluco-4-enepyranosyluronic acid)-D-galactose
ΔDi-HA: unsaturated disaccharides of hyaluronic acid, 2-acetamido-2-deoxy-3-*O*-(β-D-gluco-4-enepyranolyluronic acid)-D-glucose
ΔDi-UA-2S: unsaturated chondroitin disaccharide, 2-acetamido-2-deoxy-3-*O*-(2-*O-*sulfo-β-D-gluco-4-enepyranosyluronic acid)-D-galactose
